# Crystal structure of bis­(*N*-methyl-*N*-phenyl­amino)­tris­ulfane

**DOI:** 10.1107/S2056989015011342

**Published:** 2015-06-24

**Authors:** George Barany, Matthew J. Henley, Lauren A. Polski, Alayne L. Schroll, Victor G. Young

**Affiliations:** a Department of Chemistry, University of Minnesota, Minneapolis, MN 55455, USA; b Department of Chemistry, Saint Michael’s College, Colchester, VT 05439, USA

**Keywords:** crystal structure, tris­ulfane, organosulfur compounds, C—H⋯π inter­actions

## Abstract

The title compound, C_14_H_16_N_2_S_3_, crystallized with two independent mol­ecules [(**1** ***a***) and (**1** ***b***)] in the asymmetric unit. Both mol­ecules display a pseudo-*trans* conformation. The two consecutive S—S bond lengths of the tris­ulfane unit of mol­ecule (**1** ***a***) are 2.06 (3) and 2.08 (3) Å, and 2.08 (3) and 2.07 (2) Å for mol­ecule (**1** ***b***). Torsion angles about each of the two S—S bonds are 86.6 (2) and 87.0 (2)° for (**1** ***a***), and −84.6 (2) and −85.9 (2)° for (**1** ***b***). The core atoms, *viz.* the N—S—S—S—N moiety, of the two mol­ecules superimpose well if one is inverted on the other, but the phenyl groups do not. Thus, the two units are essentially conformational enanti­omers. In mol­ecule (**1** ***a***), the two phenyl rings are inclined to one another by 86.7 (3)°, and in mol­ecule (**1** ***b***), by 81.1 (3)°. In the crystal, mol­ecules are linked *via* C—H⋯π inter­actions, forming sheets lying parallel to (010).

## Abstract 2

The title compound was obtained in crystalline form after preparative HPLC. Conformation of the proposed mol­ecular structure was obtained by single-crystal X-ray analysis at 173 K. The mol­ecules do not take advantage of the twofold axis provided as an available symmetry option by the *Fdd*2 space group. Instead, there are two mol­ecules in the asymmetric unit, and both of them display a pseudo-*trans* conformation.

## Chemical context   

The reactions of substrates with one or two sulfanyl chloride, acid chloride, and/or (alk­oxy­dichloro­meth­yl)sulfanyl moieties have been of inter­est to our laboratory for some time (Barany *et al.*, 1983 ▸; Barany & Mott, 1984 ▸; Schroll & Barany, 1986 ▸; Schroll *et al.*, 1990 ▸; Schroll *et al.*, 2012 ▸). In some of these experiments, bis­[meth­yl(phen­yl)amino]­tris­ulfane was a component of more complicated mixtures of polysulfanes with varying numbers of S atoms. One such mixture was separated by preparative HPLC at 298 K, eluting with methanol–water (17:3). The fraction containing the title compound (dissolved in the eluting solvent) was cooled to 277 K, after which the tris­ulfane was obtained directly in crystalline form.
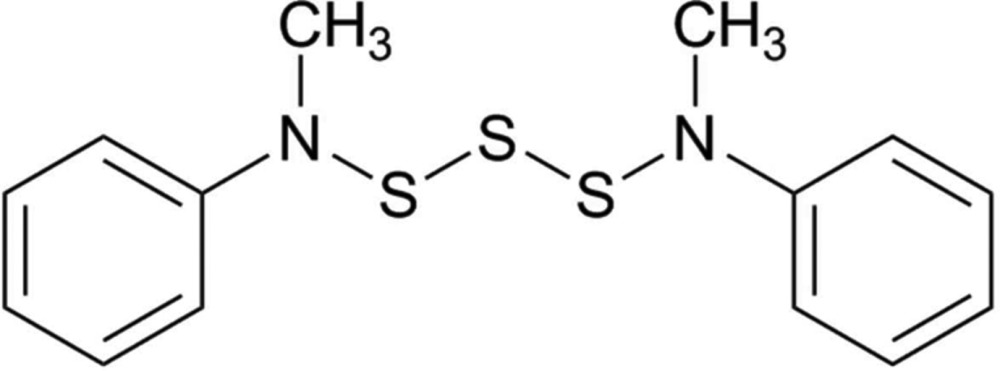


## Structural commentary   

**Figure 1:**
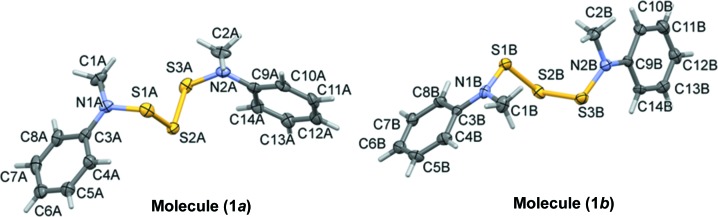
The mol­ecular structure of the title compound, showing the atom labelling. Displacement ellipsoids are drawn at the 50% probability level.

The title compound, (**1**), was obtained in crystalline form after preparative HPLC, as described by Schroll & Barany (1986 ▸). The proposed mol­ecular structure of (**1**) was confirmed by single-crystal X-ray analysis at 173 K. The mol­ecules do not take advantage of the twofold axis provided as an available symmetry option by the *Fdd*2 space group. Instead, there are two mol­ecules, (**1** ***a***) and (**1** ***b***), in the asymmetric unit (Fig. 1 ▸), and both of them display a pseudo-*trans* conformation (see later). All bond distances and angles in both mol­ecules are within expected ranges. Selected geometric parameters for compound (**1**) are given in Table 1 ▸. The two consecutive S—S bond lengths (comprising the tris­ulfane) of mol­ecule (**1** ***a***) are 2.064 (3) and 2.078 (3) Å, and for mol­ecule (**1** ***b***) are 2.076 (3) and 2.067 (2) Å. These values are similar to the value of 2.07 Å reported for the S—S bond length in elemental sulfur (S_8_). Torsion angles about each of the two S—S bonds (comprising the tris­ulfane) are, respectively, 86.6 (2) and 87.0 (2)° for (**1** ***a***), and −84.6 (2) and −85.9 (2)° for (**1** ***b***). The core atoms, *viz.* the N—S—S—S—N moiety, of the two units superimpose well if one is inverted on the other, but the phenyl groups do not. Thus, the two units are essentially conformational enanti­omers. Moreover, with respect to the four measured torsion angles, which range in absolute value from 84.6 (2) to 87.0 (2)°, these are slightly smaller than the theoretical optimum of 90.0° (Pauling, 1949 ▸; Torrico-Vallejos *et al.*, 2010 ▸). Finally, given the presence of three consecutive linearly connected sulfur atoms, representing two dihedral angles close to 90°, it is noteworthy that both of the mol­ecules in the asymmetric unit display a pseudo-*trans* conformation (torsion angles +,+ or -,- across the two S—S bonds). The theoretically possible pseudo-*cis* (torsion angles +,- or -,+) conformation (Meyer, 1976 ▸) was not observed for these structures.

## Supra­molecular features   

**Figure 2:**
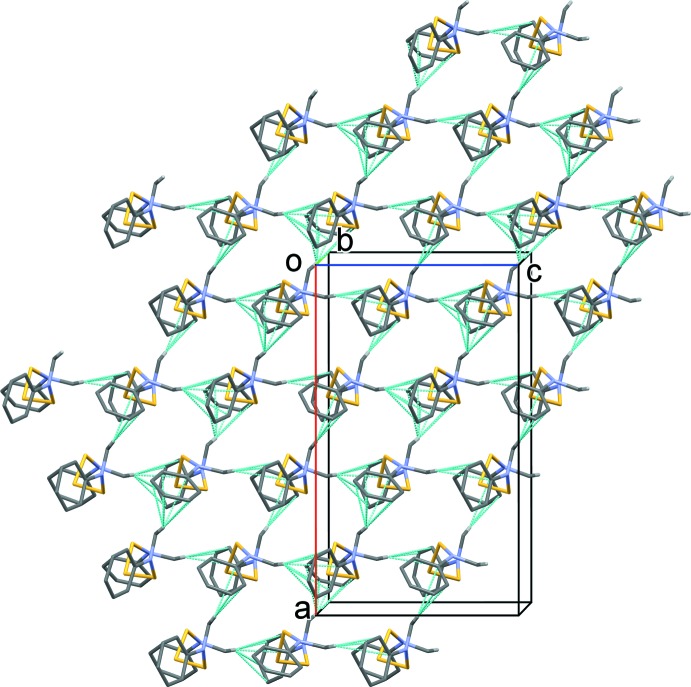
A view along the *b* axis of the crystal packing of the title compound. The dashed lines indicate the C—H⋯π inter­actions (see Table 2 ▸ for details). Only the H atoms involved in these inter­actions have been included for clarity.

In the crystal of (**1**), mol­ecules are linked *via* C—H⋯π inter­actions, forming sheets lying parallel to (010) (see Table 2 ▸ and Fig. 2 ▸).

## Database survey   

**Figure 3:**
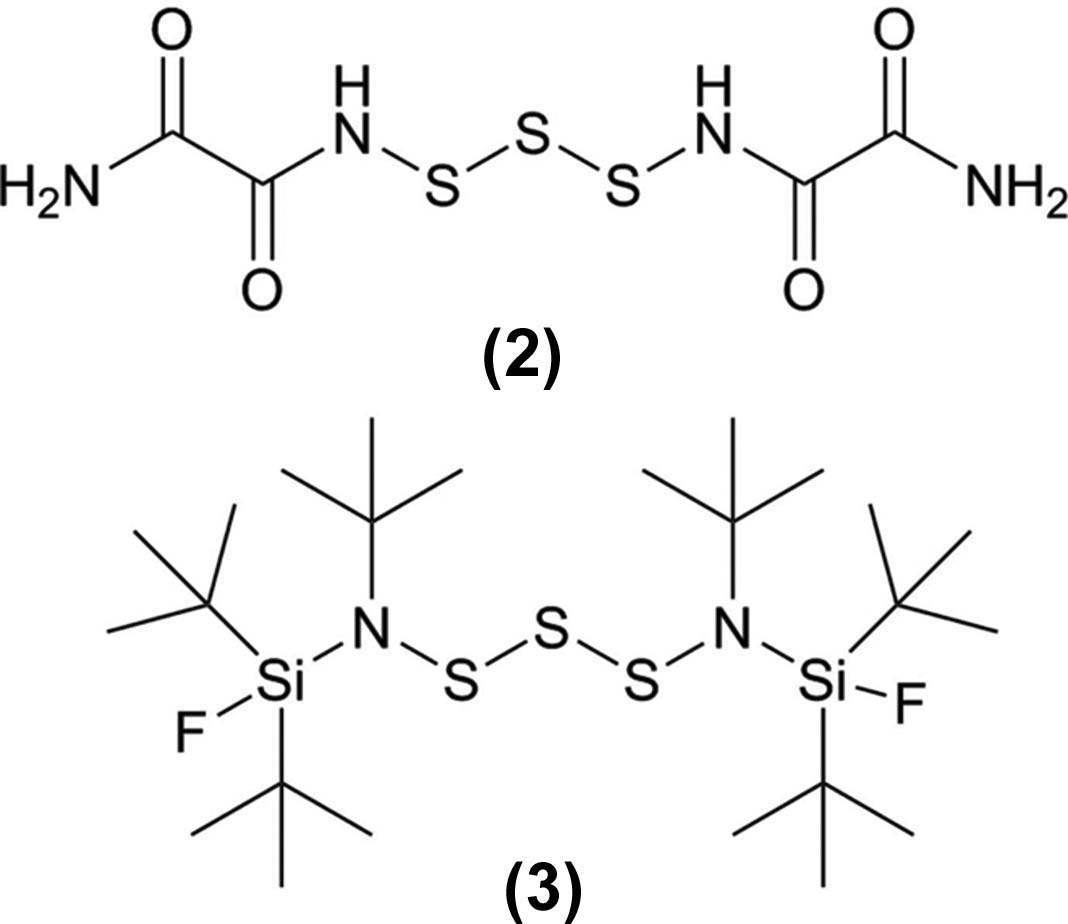
Compounds that also have an N—S—S—S—N moiety, *viz.* bis­(oxamido)­tris­ulfane, (**2**) (CSD refcode, GEHPUE; Brunn *et al.*, 1988 ▸), and bis­[*tert*-but­yl(di-*tert*-butyl­fluoro­sil­yl)amino]­tris­ulfane, (**3**) (SOTLAO; Klingebiel *et al.*, 1991 ▸).

A search of the Cambridge Structural Database (CSD, Version 5.36, February 2015; Groom & Allen, 2014 ▸) revealed the presence of two compounds (see Fig. 3 ▸) that also have an N—S—S—S—N moiety, *viz.* bis­(oxamido)­tris­ulfane, (**2**) (CSD refcode GEHPUE; Brunn *et al.*, 1988 ▸), and bis­[*tert*-but­yl(di-*tert*-butyl­fluoro­sil­yl)amino]­tris­ulfane, (**3**) (SOTLAO; Klingebiel *et al.*, 1991 ▸). Unlike the title compound, (**1**), compounds (**2**) and (**3**) each have a unique conformation in the unit cell (*Z*′ = 1). Selected geometric parameters of (**1**) and the comparison compounds, (**2**) and (**3**), are given in Table 1 ▸. While the average S—S bond length of the title compound is *ca* 2.07 Å, the corresponding value is longer (2.09 Å) in (**3**) and shorter (2.04 Å) in (**2**). The absolute value of the average torsion angle of the title compound (**1**) is *ca* 86.0°, while the corresponding value is larger (93.2 and −89.5°) and closer to the theoretical optimum in (**2**), and significantly larger (109.7 and 95.9°) in (**3**).

*Note regarding nomenclature*: In the discussion above, a consistent nomenclature scheme has been used that differs from the names used in the original publications, *viz.* bis(oxamido)­tris­ulfan, (**2**) (Brunn *et al.*, 1988 ▸) and 1,3-bis­[*tert*-but­yl(di-*tert*-butyl­fluorsil­yl)amino]­tris­ulfan, (**3**) (Klingebiel *et al.*, 1991 ▸).

## Synthesis and crystallization   

The title compound, (**1**), was synthesized and obtained in crystalline form after preparative HPLC, as described by Schroll & Barany (1986 ▸): compound (**37**) in that publication.

## Refinement   

Crystal data, data collection and structure refinement details are summarized in Table 3 ▸. The H atoms were positioned geometrically and refined using a riding model, with C—H = 0.95–0.98 Å and *U*_iso_(H) = 1.5*U*_eq_(C) for methyl H atoms and 1.2*U*_eq_(C) for other H atoms.

## Supplementary Material

Crystal structure: contains datablock(s) I, Global. DOI: 10.1107/S2056989015011342/su5144sup1.cif

Structure factors: contains datablock(s) I. DOI: 10.1107/S2056989015011342/su5144Isup2.hkl

Supporting information file. DOI: 10.1107/S2056989015011342/su5144Isup3.cml

CCDC reference: 1406065

Additional supporting information:  crystallographic information; 3D view; checkCIF report

## Figures and Tables

**Table 1:** Selected geometric parameters (, ) of the title compound (**1**), and the comparison compounds (**2**) and (**3**)

	(**1*a***)	(**1*b***)	(**2**)	(**3**)
S1N1	1.664(5)	1.653(5)	1.693(2)	1.668(2)
S1S2	2.064(3)	2.076(3)	2.040(1)	2.102(1)
S2S3	2.078(3)	2.067(2)	2.045(1)	2.082(1)
S3N2	1.663(6)	1.649(5)	1.687(2)	1.680(2)
				
N1S1S2	106.9(2)	107.3(2)	105.0(1)	110.0(1)
S1S2S3	106.05(11)	105.41(11)	105.2(2)	104.7(1)
N2S3S2	107.6(2)	107.2(2)	103.8(1)	110.3(1)
				
N1S1S2S3	86.6(2)	84.6(2)	93.2(7)	109.7(2)
S1S2S3N2	87.0(2)	85.9(2)	89.5(2)	95.9(1)

**Table 2:** Hydrogen-bond geometry (, ) *Cg*1, *Cg*2, *Cg*3, and *Cg*4 are the centroids of rings C3*A*C8*A*, C9*A*C14*A*, C3*B*C8*B*, and C9*B*C14*B*, respectively.

*D*H*A*	*D*H	H*A*	*D* *A*	*D*H*A*
C1*A*H1*AA* *Cg*2^i^	0.98	2.91	3.810(7)	153
C2*A*H2*AA* *Cg*3^ii^	0.98	2.76	3.658(8)	153
C1*B*H1*BA* *Cg*4^iii^	0.98	2.73	3.575(7)	145
C2*B*H2*BA* *Cg*1^ii^	0.98	2.98	3.870(7)	151

Symmetry codes: (i) *x* 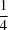, *y*+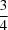, *z*+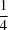; (ii) *x*+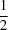, *y*+1, *z*+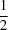; (iii) *x*+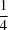, *y*+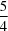, *z*+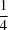.

**Table 3:** Experimental details

Crystal data	
Chemical formula	C_14_H_16_N_2_S_3_
*M*_r_	308.47
Crystal system, space group	Orthorhombic, *F* *d* *d*2
Temperature (K)	173
*a*, *b*, *c* ()	19.284(3), 56.440(8), 11.1695(15)
*V* (^3^)	12157(3)
*Z*	32
Radiation type	Mo *K*
(mm^1^)	0.48
Crystal size (mm)	0.25 0.22 0.04

Data collection	
Diffractometer	Bruker *SMART* CCD area detector
Absorption correction	Multi-scan (*SADABS*; Bruker, 2001 ▸)
*T*_min_, *T*_max_	0.890, 0.981
No. of measured, independent and observed [*I* > 2(*I*)] reflections	15884, 4978, 3097
*R*_int_	0.075
(sin /)_max_ (^1^)	0.597

Refinement	
*R*[*F* ^2^ > 2(*F* ^2^)], *wR*(*F* ^2^), *S*	0.056, 0.129, 1.06
No. of reflections	4978
No. of parameters	347
No. of restraints	1
H-atom treatment	H-atom parameters constrained
	*w* = 1/[^2^(*F*_o_^2^) + (0.0357*P*)^2^ + 36.8709*P*] where *P* = (*F*_o_^2^ + 2*F*_c_^2^)/3
_max_, _min_ (e ^3^)	0.43, 0.31
Absolute structure	2194 Friedel pairs (Flack, 1983 ▸)
Absolute structure parameter	0.08(12)

Computer programs: *SMART* and *SAINT* (Bruker, 2001 ▸), *SHELXS97* and *SHELXL97* (Sheldrick, 2008 ▸) and *Mercury* (Macrae *et al.*, 2008 ▸).

## supplementary crystallographic information

### Crystal data

**Table:**

C_14_H_16_N_2_S_3_	*D*_x_ = 1.348 Mg m^−^^3^
*M**_r_* = 308.47	Melting point: 353 K
Orthorhombic, *F**d**d*2	Mo *K*α radiation, λ = 0.71073 Å
Hall symbol: F 2 -2d	Cell parameters from 1945 reflections
*a* = 19.284 (3) Å	θ = 2.4–24.9°
*b* = 56.440 (8) Å	µ = 0.48 mm^−^^1^
*c* = 11.1695 (15) Å	*T* = 173 K
*V* = 12157 (3) Å^3^	Plate, colorless
*Z* = 32	0.25 × 0.22 × 0.04 mm
*F*(000) = 5184	

### Data collection

**Table:**

Bruker SMART CCD area-detector diffractometer	4978 independent reflections
Radiation source: sealed tube	3097 reflections with *I* > 2σ(*I*)
Graphite monochromator	*R*_int_ = 0.075
φ and ω scans	θ_max_ = 25.1°, θ_min_ = 1.4°
Absorption correction: multi-scan (*SADABS*; Bruker, 2001)	*h* = 0→22
*T*_min_ = 0.890, *T*_max_ = 0.981	*k* = 0→67
15884 measured reflections	*l* = −13→11

### Refinement

**Table:**

Refinement on *F*^2^	Secondary atom site location: difference Fourier map
Least-squares matrix: full	Hydrogen site location: inferred from neighbouring sites
*R*[*F*^2^ > 2σ(*F*^2^)] = 0.056	H-atom parameters constrained
*wR*(*F*^2^) = 0.129	*w* = 1/[σ^2^(*F*_o_^2^) + (0.0357*P*)^2^ + 36.8709*P*] where *P* = (*F*_o_^2^ + 2*F*_c_^2^)/3
*S* = 1.06	(Δ/σ)_max_ = 0.001
4978 reflections	Δρ_max_ = 0.43 e Å^−^^3^
347 parameters	Δρ_min_ = −0.31 e Å^−^^3^
1 restraint	Absolute structure: 2194 Friedel pairs (Flack, 1983)
Primary atom site location: structure-invariant direct methods	Absolute structure parameter: 0.08 (12)

### Special details

**Table:**

Geometry. All e.s.d.'s (except the e.s.d. in the dihedral angle between two l.s. planes) are estimated using the full covariance matrix. The cell e.s.d.'s are taken into account individually in the estimation of e.s.d.'s in distances, angles and torsion angles; correlations between e.s.d.'s in cell parameters are only used when they are defined by crystal symmetry. An approximate (isotropic) treatment of cell e.s.d.'s is used for estimating e.s.d.'s involving l.s. planes.
Refinement. Refinement of *F*^2^ against all reflections. The weighted *R*-factor *wR* and goodness of fit *S* are based on *F*^2^, conventional *R*-factors *R* are based on *F*, with *F* set to zero for negative *F*^2^. The threshold expression of *F*^2^ > 2σ(*F*^2^) is used only for calculating *R*-factors(gt) *etc*. and is not relevant to the choice of reflections for refinement. *R*-factors based on *F*^2^ are statistically about twice as large as those based on *F*, and *R*-factors based on all data will be even larger.

### Fractional atomic coordinates and isotropic or equivalent isotropic displacement parameters (Å^2^)

**Table:**

	*x*	*y*	*z*	*U*_iso_*/*U*_eq_	
S1A	0.16203 (9)	0.35562 (3)	0.42989 (17)	0.0509 (5)	
S2A	0.13888 (9)	0.37674 (3)	0.28446 (18)	0.0512 (5)	
S3A	0.05789 (9)	0.39854 (3)	0.33839 (17)	0.0529 (5)	
N1A	0.1096 (2)	0.33225 (10)	0.4230 (5)	0.0430 (14)	
N2A	0.0925 (3)	0.42227 (10)	0.4033 (5)	0.0460 (14)	
C1A	0.0371 (3)	0.33682 (13)	0.4515 (6)	0.0555 (19)	
H1AA	0.0155	0.3222	0.4809	0.083*	
H1AB	0.0129	0.3422	0.3794	0.083*	
H1AC	0.0343	0.3491	0.5133	0.083*	
C2A	0.1179 (4)	0.41859 (13)	0.5256 (6)	0.063 (2)	
H2AA	0.1143	0.4334	0.5706	0.095*	
H2AB	0.1665	0.4135	0.5230	0.095*	
H2AC	0.0899	0.4064	0.5649	0.095*	
C3A	0.1249 (3)	0.31310 (11)	0.3459 (6)	0.0428 (18)	
C4A	0.1941 (3)	0.30690 (11)	0.3200 (6)	0.0501 (19)	
H4AA	0.2307	0.3164	0.3502	0.060*	
C5A	0.2096 (4)	0.28734 (13)	0.2520 (7)	0.059 (2)	
H5AA	0.2567	0.2832	0.2383	0.071*	
C6A	0.1577 (4)	0.27368 (13)	0.2037 (7)	0.056 (2)	
H6AA	0.1684	0.2605	0.1544	0.067*	
C7A	0.0894 (5)	0.27962 (12)	0.2284 (7)	0.063 (2)	
H7AA	0.0531	0.2699	0.1980	0.076*	
C8A	0.0733 (3)	0.29903 (12)	0.2953 (7)	0.0514 (19)	
H8AA	0.0260	0.3030	0.3076	0.062*	
C9A	0.1215 (3)	0.44089 (11)	0.3334 (7)	0.0408 (17)	
C10A	0.1734 (3)	0.45591 (12)	0.3798 (6)	0.0458 (19)	
H10A	0.1906	0.4533	0.4584	0.055*	
C11A	0.1992 (3)	0.47418 (11)	0.3132 (7)	0.052 (2)	
H11A	0.2340	0.4841	0.3462	0.062*	
C12A	0.1756 (4)	0.47837 (12)	0.2001 (8)	0.055 (2)	
H12A	0.1935	0.4912	0.1542	0.066*	
C13A	0.1253 (4)	0.46368 (12)	0.1536 (7)	0.055 (2)	
H13A	0.1086	0.4665	0.0749	0.066*	
C14A	0.0987 (4)	0.44514 (12)	0.2171 (7)	0.0514 (19)	
H14A	0.0646	0.4352	0.1821	0.062*	
S1B	0.34517 (9)	0.60013 (3)	0.43382 (17)	0.0503 (5)	
S2B	0.35517 (9)	0.62207 (3)	0.28572 (18)	0.0504 (5)	
S3B	0.44058 (9)	0.64310 (3)	0.32075 (16)	0.0473 (5)	
N1B	0.3972 (2)	0.57722 (9)	0.4127 (5)	0.0406 (13)	
N2B	0.4133 (3)	0.66599 (9)	0.3995 (5)	0.0410 (14)	
C1B	0.4706 (3)	0.58075 (12)	0.4394 (7)	0.056 (2)	
H1BA	0.4893	0.5665	0.4775	0.084*	
H1BB	0.4758	0.5943	0.4936	0.084*	
H1BC	0.4958	0.5839	0.3649	0.084*	
C2B	0.3957 (4)	0.66091 (11)	0.5245 (6)	0.0502 (18)	
H2BA	0.4010	0.6754	0.5724	0.075*	
H2BB	0.4269	0.6486	0.5554	0.075*	
H2BC	0.3477	0.6554	0.5293	0.075*	
C3B	0.3785 (3)	0.55805 (10)	0.3387 (6)	0.0360 (15)	
C4B	0.4264 (3)	0.54146 (11)	0.2998 (6)	0.0470 (18)	
H4BA	0.4739	0.5435	0.3201	0.056*	
C5B	0.4072 (4)	0.52205 (12)	0.2322 (6)	0.0528 (19)	
H5BA	0.4413	0.5108	0.2098	0.063*	
C6B	0.3392 (4)	0.51874 (12)	0.1965 (7)	0.052 (2)	
H6BA	0.3259	0.5056	0.1486	0.062*	
C7B	0.2916 (3)	0.53537 (11)	0.2336 (6)	0.0467 (18)	
H7BA	0.2444	0.5335	0.2110	0.056*	
C8B	0.3098 (3)	0.55452 (10)	0.3020 (6)	0.0418 (16)	
H8BA	0.2753	0.5656	0.3249	0.050*	
C9B	0.3837 (3)	0.68621 (10)	0.3444 (7)	0.0369 (16)	
C10B	0.3412 (3)	0.70186 (10)	0.4077 (6)	0.0446 (18)	
H10B	0.3288	0.6984	0.4881	0.054*	
C11B	0.3175 (4)	0.72197 (13)	0.3558 (8)	0.060 (2)	
H11B	0.2898	0.7326	0.4015	0.072*	
C12B	0.3328 (4)	0.72757 (12)	0.2363 (8)	0.054 (2)	
H12B	0.3158	0.7417	0.2003	0.065*	
C13B	0.3730 (3)	0.71203 (11)	0.1735 (7)	0.0481 (17)	
H13B	0.3840	0.7153	0.0922	0.058*	
C14B	0.3981 (3)	0.69158 (11)	0.2259 (7)	0.0436 (17)	
H14B	0.4257	0.6810	0.1800	0.052*	

### Atomic displacement parameters (Å^2^)

**Table:**

	*U*^11^	*U*^22^	*U*^33^	*U*^12^	*U*^13^	*U*^23^
S1A	0.0414 (11)	0.0603 (11)	0.0510 (14)	0.0036 (8)	−0.0136 (9)	−0.0069 (10)
S2A	0.0612 (11)	0.0511 (10)	0.0414 (12)	−0.0137 (8)	0.0091 (10)	−0.0026 (10)
S3A	0.0373 (10)	0.0635 (11)	0.0577 (14)	−0.0058 (8)	−0.0102 (9)	0.0142 (10)
N1A	0.032 (3)	0.054 (3)	0.043 (4)	0.006 (2)	−0.002 (3)	0.008 (3)
N2A	0.049 (3)	0.054 (4)	0.035 (4)	0.005 (3)	0.000 (3)	0.001 (3)
C1A	0.029 (4)	0.085 (5)	0.053 (5)	0.000 (3)	0.002 (3)	0.019 (4)
C2A	0.070 (5)	0.090 (5)	0.029 (5)	0.029 (4)	−0.007 (4)	−0.003 (4)
C3A	0.033 (4)	0.050 (4)	0.046 (5)	−0.006 (3)	−0.005 (3)	0.017 (4)
C4A	0.038 (4)	0.055 (4)	0.057 (6)	−0.003 (3)	−0.008 (4)	−0.005 (4)
C5A	0.058 (5)	0.057 (5)	0.062 (6)	−0.004 (4)	0.001 (4)	0.000 (4)
C6A	0.072 (6)	0.055 (5)	0.041 (5)	−0.008 (4)	−0.002 (4)	0.006 (3)
C7A	0.084 (6)	0.049 (5)	0.056 (6)	−0.024 (4)	−0.029 (4)	0.018 (4)
C8A	0.042 (4)	0.053 (4)	0.060 (6)	−0.010 (3)	−0.015 (4)	0.014 (4)
C9A	0.030 (4)	0.049 (4)	0.043 (5)	0.014 (3)	−0.001 (3)	−0.005 (3)
C10A	0.042 (4)	0.059 (4)	0.037 (5)	0.011 (3)	−0.013 (3)	−0.018 (3)
C11A	0.047 (4)	0.035 (4)	0.074 (7)	0.001 (3)	−0.012 (4)	−0.018 (4)
C12A	0.061 (5)	0.035 (4)	0.069 (6)	0.005 (3)	0.007 (4)	−0.002 (4)
C13A	0.067 (5)	0.050 (4)	0.050 (6)	0.002 (4)	−0.012 (4)	0.000 (4)
C14A	0.052 (4)	0.050 (4)	0.052 (6)	−0.005 (3)	−0.019 (4)	−0.004 (4)
S1B	0.0489 (11)	0.0517 (10)	0.0502 (14)	−0.0096 (8)	0.0123 (10)	−0.0097 (9)
S2B	0.0595 (11)	0.0440 (10)	0.0476 (12)	0.0073 (8)	−0.0123 (10)	−0.0093 (9)
S3B	0.0400 (10)	0.0478 (10)	0.0540 (14)	0.0062 (7)	0.0123 (9)	0.0058 (8)
N1B	0.039 (3)	0.041 (3)	0.041 (4)	−0.011 (2)	−0.004 (3)	0.005 (3)
N2B	0.039 (3)	0.045 (3)	0.039 (4)	−0.004 (2)	−0.002 (3)	−0.002 (3)
C1B	0.045 (4)	0.061 (4)	0.062 (6)	−0.011 (3)	−0.016 (4)	0.009 (4)
C2B	0.057 (4)	0.061 (4)	0.033 (5)	−0.014 (3)	−0.003 (4)	0.002 (4)
C3B	0.039 (4)	0.040 (4)	0.028 (4)	−0.007 (3)	0.004 (3)	0.007 (3)
C4B	0.037 (4)	0.057 (4)	0.047 (5)	0.013 (3)	0.000 (4)	0.009 (4)
C5B	0.050 (5)	0.056 (4)	0.052 (5)	0.016 (3)	0.002 (4)	−0.008 (4)
C6B	0.060 (5)	0.047 (4)	0.049 (6)	0.001 (3)	0.006 (4)	−0.001 (3)
C7B	0.041 (4)	0.050 (4)	0.049 (5)	−0.012 (3)	−0.001 (3)	−0.005 (3)
C8B	0.034 (4)	0.045 (4)	0.047 (5)	0.004 (3)	0.001 (3)	−0.002 (3)
C9B	0.029 (3)	0.037 (4)	0.045 (5)	−0.007 (3)	0.002 (3)	−0.014 (3)
C10B	0.051 (4)	0.045 (4)	0.037 (5)	−0.004 (3)	0.001 (3)	−0.005 (3)
C11B	0.061 (5)	0.048 (5)	0.071 (7)	0.003 (4)	0.000 (5)	−0.015 (4)
C12B	0.051 (4)	0.041 (4)	0.071 (6)	0.004 (3)	−0.013 (4)	−0.007 (4)
C13B	0.042 (4)	0.057 (4)	0.045 (5)	−0.002 (3)	0.005 (4)	0.005 (4)
C14B	0.044 (4)	0.042 (4)	0.044 (5)	0.004 (3)	0.017 (3)	−0.006 (3)

### Geometric parameters (Å, º)

**Table:**

S1A—N1A	1.664 (5)	S1B—N1B	1.653 (5)
S1A—S2A	2.064 (3)	S1B—S2B	2.076 (3)
S2A—S3A	2.078 (3)	S2B—S3B	2.067 (2)
S3A—N2A	1.663 (6)	S3B—N2B	1.649 (5)
N1A—C3A	1.413 (8)	N1B—C3B	1.408 (8)
N1A—C1A	1.457 (7)	N1B—C1B	1.460 (7)
N2A—C9A	1.424 (8)	N2B—C9B	1.416 (8)
N2A—C2A	1.465 (8)	N2B—C2B	1.465 (8)
C1A—H1AA	0.9800	C1B—H1BA	0.9800
C1A—H1AB	0.9800	C1B—H1BB	0.9800
C1A—H1AC	0.9800	C1B—H1BC	0.9800
C2A—H2AA	0.9800	C2B—H2BA	0.9800
C2A—H2AB	0.9800	C2B—H2BB	0.9800
C2A—H2AC	0.9800	C2B—H2BC	0.9800
C3A—C8A	1.393 (8)	C3B—C4B	1.386 (8)
C3A—C4A	1.410 (9)	C3B—C8B	1.401 (8)
C4A—C5A	1.373 (9)	C4B—C5B	1.381 (9)
C4A—H4AA	0.9500	C4B—H4BA	0.9500
C5A—C6A	1.373 (9)	C5B—C6B	1.383 (9)
C5A—H5AA	0.9500	C5B—H5BA	0.9500
C6A—C7A	1.388 (10)	C6B—C7B	1.377 (9)
C6A—H6AA	0.9500	C6B—H6BA	0.9500
C7A—C8A	1.362 (10)	C7B—C8B	1.369 (8)
C7A—H7AA	0.9500	C7B—H7BA	0.9500
C8A—H8AA	0.9500	C8B—H8BA	0.9500
C9A—C14A	1.392 (9)	C9B—C14B	1.386 (9)
C9A—C10A	1.410 (9)	C9B—C10B	1.396 (8)
C10A—C11A	1.365 (9)	C10B—C11B	1.354 (9)
C10A—H10A	0.9500	C10B—H10B	0.9500
C11A—C12A	1.363 (10)	C11B—C12B	1.403 (10)
C11A—H11A	0.9500	C11B—H11B	0.9500
C12A—C13A	1.378 (9)	C12B—C13B	1.365 (9)
C12A—H12A	0.9500	C12B—H12B	0.9500
C13A—C14A	1.365 (9)	C13B—C14B	1.382 (9)
C13A—H13A	0.9500	C13B—H13B	0.9500
C14A—H14A	0.9500	C14B—H14B	0.9500
			
N1A—S1A—S2A	106.9 (2)	N1B—S1B—S2B	107.3 (2)
S1A—S2A—S3A	106.05 (11)	S3B—S2B—S1B	105.41 (11)
N2A—S3A—S2A	107.6 (2)	N2B—S3B—S2B	107.2 (2)
C3A—N1A—C1A	118.0 (5)	C3B—N1B—C1B	118.2 (5)
C3A—N1A—S1A	120.5 (4)	C3B—N1B—S1B	122.0 (4)
C1A—N1A—S1A	115.6 (4)	C1B—N1B—S1B	116.9 (4)
C9A—N2A—C2A	119.0 (6)	C9B—N2B—C2B	118.6 (5)
C9A—N2A—S3A	120.9 (5)	C9B—N2B—S3B	121.9 (5)
C2A—N2A—S3A	115.3 (5)	C2B—N2B—S3B	115.4 (4)
N1A—C1A—H1AA	109.5	N1B—C1B—H1BA	109.5
N1A—C1A—H1AB	109.5	N1B—C1B—H1BB	109.5
H1AA—C1A—H1AB	109.5	H1BA—C1B—H1BB	109.5
N1A—C1A—H1AC	109.5	N1B—C1B—H1BC	109.5
H1AA—C1A—H1AC	109.5	H1BA—C1B—H1BC	109.5
H1AB—C1A—H1AC	109.5	H1BB—C1B—H1BC	109.5
N2A—C2A—H2AA	109.5	N2B—C2B—H2BA	109.5
N2A—C2A—H2AB	109.5	N2B—C2B—H2BB	109.5
H2AA—C2A—H2AB	109.5	H2BA—C2B—H2BB	109.5
N2A—C2A—H2AC	109.5	N2B—C2B—H2BC	109.5
H2AA—C2A—H2AC	109.5	H2BA—C2B—H2BC	109.5
H2AB—C2A—H2AC	109.5	H2BB—C2B—H2BC	109.5
C8A—C3A—C4A	116.9 (7)	C4B—C3B—C8B	116.3 (6)
C8A—C3A—N1A	122.3 (6)	C4B—C3B—N1B	122.2 (6)
C4A—C3A—N1A	120.8 (6)	C8B—C3B—N1B	121.5 (6)
C5A—C4A—C3A	121.2 (6)	C5B—C4B—C3B	121.8 (6)
C5A—C4A—H4AA	119.4	C5B—C4B—H4BA	119.1
C3A—C4A—H4AA	119.4	C3B—C4B—H4BA	119.1
C4A—C5A—C6A	120.7 (7)	C4B—C5B—C6B	121.3 (6)
C4A—C5A—H5AA	119.6	C4B—C5B—H5BA	119.3
C6A—C5A—H5AA	119.6	C6B—C5B—H5BA	119.3
C5A—C6A—C7A	118.6 (8)	C7B—C6B—C5B	116.9 (7)
C5A—C6A—H6AA	120.7	C7B—C6B—H6BA	121.5
C7A—C6A—H6AA	120.7	C5B—C6B—H6BA	121.5
C8A—C7A—C6A	121.3 (7)	C8B—C7B—C6B	122.3 (6)
C8A—C7A—H7AA	119.3	C8B—C7B—H7BA	118.8
C6A—C7A—H7AA	119.3	C6B—C7B—H7BA	118.8
C7A—C8A—C3A	121.2 (7)	C7B—C8B—C3B	121.2 (6)
C7A—C8A—H8AA	119.4	C7B—C8B—H8BA	119.4
C3A—C8A—H8AA	119.4	C3B—C8B—H8BA	119.4
C14A—C9A—C10A	117.7 (6)	C14B—C9B—C10B	117.5 (6)
C14A—C9A—N2A	121.0 (6)	C14B—C9B—N2B	120.7 (6)
C10A—C9A—N2A	121.4 (6)	C10B—C9B—N2B	121.8 (7)
C11A—C10A—C9A	120.8 (7)	C11B—C10B—C9B	120.8 (7)
C11A—C10A—H10A	119.6	C11B—C10B—H10B	119.6
C9A—C10A—H10A	119.6	C9B—C10B—H10B	119.6
C12A—C11A—C10A	121.0 (7)	C10B—C11B—C12B	121.6 (7)
C12A—C11A—H11A	119.5	C10B—C11B—H11B	119.2
C10A—C11A—H11A	119.5	C12B—C11B—H11B	119.2
C11A—C12A—C13A	118.6 (7)	C13B—C12B—C11B	117.6 (7)
C11A—C12A—H12A	120.7	C13B—C12B—H12B	121.2
C13A—C12A—H12A	120.7	C11B—C12B—H12B	121.2
C14A—C13A—C12A	122.1 (7)	C12B—C13B—C14B	121.2 (7)
C14A—C13A—H13A	119.0	C12B—C13B—H13B	119.4
C12A—C13A—H13A	119.0	C14B—C13B—H13B	119.4
C13A—C14A—C9A	119.8 (7)	C13B—C14B—C9B	121.2 (6)
C13A—C14A—H14A	120.1	C13B—C14B—H14B	119.4
C9A—C14A—H14A	120.1	C9B—C14B—H14B	119.4
			
N1A—S1A—S2A—S3A	86.6 (2)	N1B—S1B—S2B—S3B	−84.6 (2)
S1A—S2A—S3A—N2A	87.0 (2)	S1B—S2B—S3B—N2B	−85.9 (2)
S2A—S1A—N1A—C3A	80.2 (5)	S2B—S1B—N1B—C3B	−79.9 (5)
S2A—S1A—N1A—C1A	−72.2 (5)	S2B—S1B—N1B—C1B	80.3 (5)
S2A—S3A—N2A—C9A	77.9 (5)	S2B—S3B—N2B—C9B	−83.1 (5)
S2A—S3A—N2A—C2A	−77.0 (5)	S2B—S3B—N2B—C2B	73.7 (5)
C1A—N1A—C3A—C8A	1.3 (9)	C1B—N1B—C3B—C4B	5.8 (9)
S1A—N1A—C3A—C8A	−150.5 (5)	S1B—N1B—C3B—C4B	165.7 (5)
C1A—N1A—C3A—C4A	−176.4 (6)	C1B—N1B—C3B—C8B	−175.2 (6)
S1A—N1A—C3A—C4A	31.7 (8)	S1B—N1B—C3B—C8B	−15.3 (8)
C8A—C3A—C4A—C5A	−2.5 (10)	C8B—C3B—C4B—C5B	−2.3 (10)
N1A—C3A—C4A—C5A	175.4 (6)	N1B—C3B—C4B—C5B	176.7 (6)
C3A—C4A—C5A—C6A	2.3 (12)	C3B—C4B—C5B—C6B	2.4 (11)
C4A—C5A—C6A—C7A	−2.2 (11)	C4B—C5B—C6B—C7B	−1.4 (11)
C5A—C6A—C7A—C8A	2.5 (11)	C5B—C6B—C7B—C8B	0.5 (11)
C6A—C7A—C8A—C3A	−2.8 (11)	C6B—C7B—C8B—C3B	−0.6 (11)
C4A—C3A—C8A—C7A	2.8 (10)	C4B—C3B—C8B—C7B	1.4 (10)
N1A—C3A—C8A—C7A	−175.1 (6)	N1B—C3B—C8B—C7B	−177.6 (6)
C2A—N2A—C9A—C14A	−179.5 (6)	C2B—N2B—C9B—C14B	−179.3 (5)
S3A—N2A—C9A—C14A	26.5 (8)	S3B—N2B—C9B—C14B	−23.2 (8)
C2A—N2A—C9A—C10A	−0.7 (8)	C2B—N2B—C9B—C10B	3.1 (9)
S3A—N2A—C9A—C10A	−154.7 (5)	S3B—N2B—C9B—C10B	159.1 (5)
C14A—C9A—C10A—C11A	1.1 (9)	C14B—C9B—C10B—C11B	−2.5 (10)
N2A—C9A—C10A—C11A	−177.8 (6)	N2B—C9B—C10B—C11B	175.2 (6)
C9A—C10A—C11A—C12A	−0.1 (10)	C9B—C10B—C11B—C12B	1.8 (11)
C10A—C11A—C12A—C13A	−0.3 (10)	C10B—C11B—C12B—C13B	−0.3 (10)
C11A—C12A—C13A—C14A	−0.2 (11)	C11B—C12B—C13B—C14B	−0.4 (10)
C12A—C13A—C14A—C9A	1.1 (11)	C12B—C13B—C14B—C9B	−0.4 (10)
C10A—C9A—C14A—C13A	−1.5 (10)	C10B—C9B—C14B—C13B	1.8 (9)
N2A—C9A—C14A—C13A	177.4 (6)	N2B—C9B—C14B—C13B	−175.9 (6)

### Hydrogen-bond geometry (Å, º)

*Cg*1, *Cg*2, *Cg*3, and *Cg*4 are the centroids of rings C3A–C8A, C9A–C14A, C3B–C8B, and C9B–C14B, respectively.

**Table:**

*D*—H···*A*	*D*—H	H···*A*	*D*···*A*	*D*—H···*A*
C1*A*—H1*AA*···*Cg*2^i^	0.98	2.91	3.810 (7)	153
C2*A*—H2*AA*···*Cg*3^ii^	0.98	2.76	3.658 (8)	153
C1*B*—H1*BA*···*Cg*4^iii^	0.98	2.73	3.575 (7)	145
C2*B*—H2*BA*···*Cg*1^ii^	0.98	2.98	3.870 (7)	151

Symmetry codes: (i) *x*−1/4, −*y*+3/4, *z*+1/4; (ii) −*x*+1/2, −*y*+1, *z*+1/2; (iii) *x*+1/4, −*y*+5/4, *z*+1/4.
